# Green nephrology: water and waste — one year in a hemodialysis unit in Northeastern Brazil and reassessment of dialyzer reprocessing from an environmental perspective

**DOI:** 10.1590/2175-8239-JBN-2025-0302en

**Published:** 2026-08-07

**Authors:** Ginivaldo Victor Ribeiro do Nascimento, Isabella Melo Soares, Elizana Carvalho Oliveira, Thiago de Sousa Coelho Porto

**Affiliations:** 1 Universidade Estadual do Piauí, Teresina, PI, Brazil.; 2 Centro Universitário UniFacid, Teresina, PI, Brazil.; 3 Centro de Terapia Renal, Teresina, PI, Brazil.

**Keywords:** Renal Dialysis, Environmental Sustainability, Environment, Recycling, Waste Products

## Abstract

**Introduction:**

Hemodialysis is one of the most water-intensive and waste-generating therapies in health care, yet its environmental impact remains poorly documented in low- and middle-income countries. This study aimed to estimate water consumption and solid waste generation in a hemodialysis unit in northeastern Brazil that reprocesses dialyzers, comparing the observed scenario with an estimated counterfactual single-use scenario.

**Methods:**

This was a single-center, retrospective observational study. Aggregate institutional data on water consumption, supply procurement, and waste mass were analyzed over a 12-month period. An estimated counterfactual single-use scenario was constructed using mathematical projections, assuming one new dialyzer per session. Carbon dioxide equivalent (CO_2_-eq) emissions were estimated using an emission factor for the incineration of clinical waste.

**Results:**

A total of 34,759 hemodialysis sessions were performed, with a mean of 223 patients/month. Total water consumption was 14.9 million liters, corresponding to 429 L/session (2.3% attributed to reprocessing). Solid waste generation was 30.9 t (0.89 kg/session), consisting of 21.6% contaminated waste, 51.5% solution containers/bottles, and 26.8% cardboard. Of all plastic waste, 70.4% was recycled and 29.6% was incinerated. In the counterfactual single-use scenario, waste generation was estimated to increase by 78.7%, incinerated plastics by 312%, and associated emissions by an additional 37.5 t of CO_2_-eq.

**Conclusion:**

The comparison of the two scenarios suggests that dialyzer reprocessing had a marginal effect on water consumption and reduced waste generation and associated emissions. These comparisons should be interpreted as environmental estimates; they do not allow causal inferences or universal recommendations.

## INTRODUCTION

Hemodialysis is one of the most water- and energy-intensive therapies in health care^1,2^. The environmental impact of this treatment has gained recognition only in recent years, in parallel with the global rise in chronic kidney disease. By 2025, the number of patients on dialysis is projected to reach 5-6 million, with a prevalence of 824 per million population^3,4^.

In many regions, water scarcity is already a reality, and water management in hemodialysis is often inadequate, largely because reverse osmosis systems discard up to two-thirds of the water supply^3,5^. Dialysis prescriptions and other procedure-related factors also influence overall consumption^3,5^.

Waste generation poses an additional burden. Less than one third of noninfectious waste is recycled, whereas infectious waste, which is sent for incineration, contributes to CO_2_ emissions and, consequently, to the greenhouse gas burden that drives climate change^3,6,7^. Dialyzer reprocessing has been proposed as one approach to reducing pollution and waste, with no consistent evidence of clinical inferiority^7,8^. Yet most published data remain partial, and few reports systematically describe waste composition or final disposal in resource-limited settings. Detailed data on resources, supplies, and waste are needed to support effective mitigation and sustainable disposal practices.

The aim of this study was to assess water consumption and waste generation over 12 months at a hemodialysis clinic in northeastern Brazil and to compare these findings with an estimated single-use dialyzer scenario.

## METHODS

This retrospective observational study was conducted at a single private hemodialysis unit in Teresina, Piauí, Brazil, affiliated with the Brazilian Unified Health System (*Sistema Único de Saúde*, SUS), through which 98.3% of patients were publicly funded. The unit operates three daily dialysis shifts, except on Sundays, and reprocesses dialyzers and blood tubing sets using an automated system. Data covering August 1, 2022, through July 30, 2023, were analyzed.

Water consumption data were obtained from the local water utility, the unit’s sole water source. The annual total encompassed hemodialysis sessions, the reverse osmosis system, equipment cleaning and disinfection, reprocessing, and other operational activities; disaggregation by activity was not possible. To estimate the water attributable to reprocessing, an additional 10 L per reused dialyzer was assumed, following Lacson and Lazarus^9^, recognizing that actual values may vary.

Information on supplies was obtained from pharmacy and warehouse records. Packaging materials were weighed and classified by type, including dialyzers, plastic packaging, and rigid plastic containers used to store concentrated solutions. Calculations for the observed scenario were based on the total number of dialyzers consumed during the study period, according to institutional records; a fixed average number of reuses was not used as a modeling parameter. Although the institutional protocol allows up to 20 reuses per unit, this variable was not used directly in the environmental estimates reported here. Data on non-recyclable waste were provided by the contracted waste management company.

An estimated counterfactual single-use scenario was also constructed. It assumed one new dialyzer and one new set of blood tubing per session, with the total number of sessions held constant. The projected additional quantities were estimated as the difference between the total number of sessions performed and the number of items consumed in the reprocessing scenario. These estimates were obtained through mathematical projections and do not correspond to direct empirical measurements.

Carbon dioxide equivalent (CO_2_-eq) emissions were estimated by multiplying the mass of incinerated waste by an emission factor of 1.80 kg CO_2_-eq/kg of waste^1^.

The number of patients was recorded monthly to calculate the annual mean. Mean water consumption and waste generation per patient/year were calculated based on the total number of sessions. Continuous data were expressed as totals and means, either per patient or per session, and categorical data as absolute numbers and percentages.

Because this study relied exclusively on aggregate institutional data, with no individual identification or access to identifiable information, it was deemed exempt from submission to a Research Ethics Committee under CNS Resolution No. 510/2016. Informed consent was not required.

## RESULTS

A total of 34,759 hemodialysis sessions were performed over 12 months (with a mean of 223 patients/month). Total water consumption was 14,920,000 L, corresponding to 66,906 L/patient/year and 429 L/session (Table 1).

**Table 1:** Water consumption, waste generation, and estimated CO_2_ emissions in a dialysis clinic in northeast Brazil: comparison between a clinic with dialyzer reuse and an estimated single-use dialyzer scenario (12 months)

Metric	Reuse (observed)	Single-use* (estimated)	Difference (single use vs. reuse)
Total water (12 months, L)	14,920,000	14,572,410	−2.3%
Average water per session (L/session)	429	419	−2.3%
Total waste (kg/year)	30,905.7	55,225.1	+78.7%
Total recycled (%) kg/year	24,223.2 (78.4)	27,714.2 (50.2)	+14.4%^δ^
Recycled plastics (kg/year)	15,920.2	15,920.2	0%
Recycled cardboard (kg/year)	8,304	11,794	+42%
Total incinerated (%) kg/year	6,682.5 (21.6)	27,510.9 (49.8)	+312%
Incinerated plastic items (number of items)	99,092	163,190	+64.7%
CO_2_ emissions* (kg CO_2_-eq/year)	12,028.5	49,535.8	+312%

Note – *Estimated values

^δ^ = difference in mass.

Solid waste generation reached 30,905.7 kg. Of this total, 21.6% was contaminated waste destined for incineration (dialyzers, bloodlines, and needles); 51.5% comprised plastic containers for concentrated solutions and saline bottles; and 26.8% was cardboard. The annual mean per patient was 138.6 kg (0.89 kg/session).

**Figure 1.**
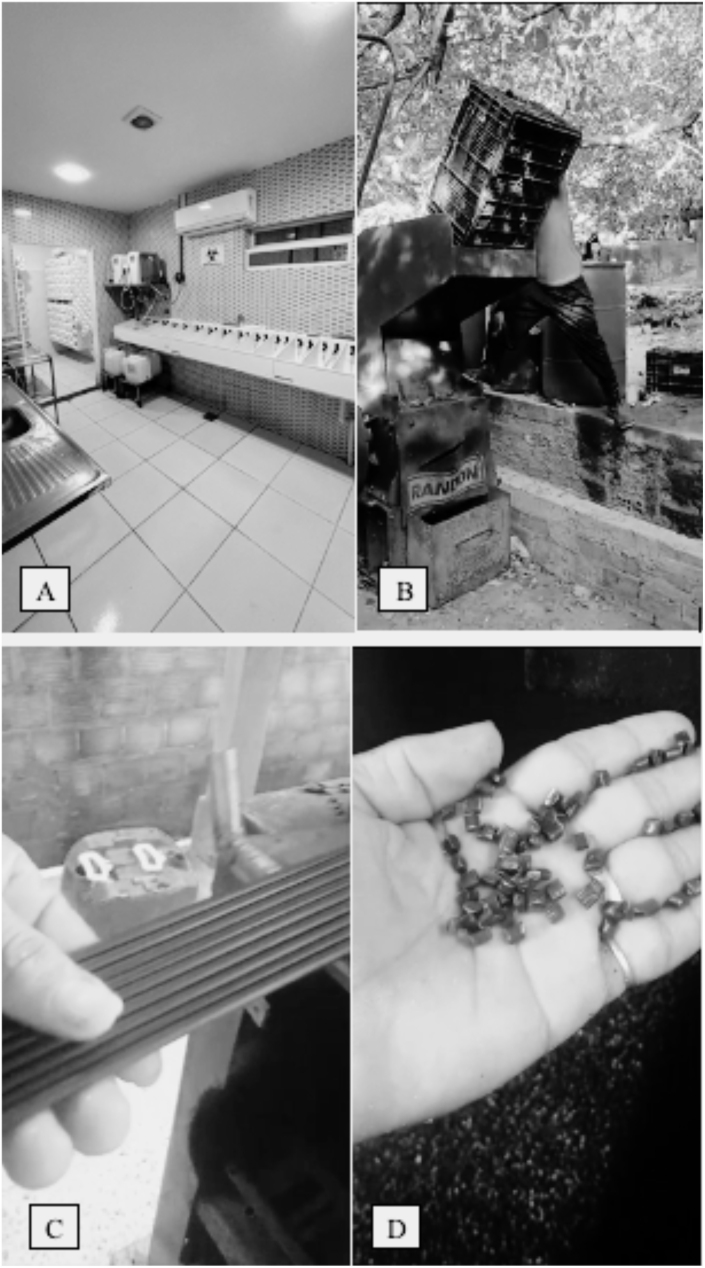
(A) Dialyzer reprocessing unit. (B) Shredding of plastic waste used in the hemodialysis clinic. (C and D) Transformation of plastic waste into raw material for manufacturing new plastic products.

Of all plastic units, 57.9% (136,724 items: saline bottles and plastic solution containers) were sent for recycling, and 42.1% were incinerated, including 57,258 needles, 2,668 blood tubing sets, 2,752 dialyzers, and 36,414 administration sets. By mass, 70.4% of plastic waste was recycled (15,920.2 kg), and 29.6% was incinerated. Recycled cardboard totaled 8,304 kg, repurposed by the automotive detergent industry, while saline bottle packaging was shredded and used as raw material for new plastic containers (Table 1; Figure 1B–D).

Over the study period, the unit generated 30.9 t of waste, and only 0.192 kg/session corresponded to non-recyclable infectious waste.

## DISCUSSION

According to the 2024 Brazilian Dialysis Census, Brazil has 172,585 patients on hemodialysis, of whom 3,412 are treated across 15 centers in Piauí, one of the country’s least socioeconomically developed states^10,11^. In this context, water recycling and single-use dialyzers are uncommon; automated reprocessing is standard practice. The data reported here thus describe not only a local experience but also a pattern that likely applies to a substantial share of Brazilian dialysis units.

**Figure 2:**
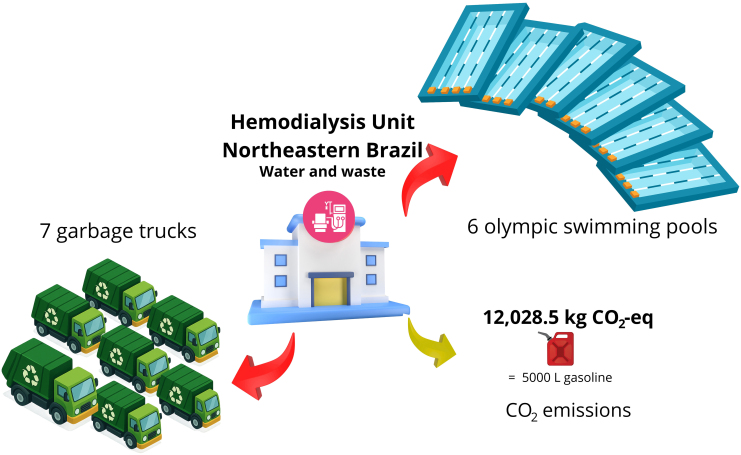
Water, waste, and estimated CO_2_ emissions — 1 year in a hemodialysis clinic in Northeastern Brazil.

International concern about climate change and health care sustainability has brought water consumption and waste generation into focus as critical challenges in hemodialysis^1,2,3,5,6,7,12,13^. In this study, annual water consumption in a single unit was 14.9 million L, enough to fill six olympic-size swimming pools or to supply a town of nearly 1,000 inhabitants for one year (Figure 2). Mean consumption per session (429 L/session) was higher than that reported by Sahay et al.^14^ (250 L) and similar to estimates widely used in the literature (500 L), which are derived from theoretical calculations considering a dialysate flow of 500 mL/min for a four-hour session plus losses related to reverse osmosis^3,6,14^. The annual consumption of 66,906 L/patient observed here was lower than the theoretical projection of 80,000 L^3,8,14^.

Dialyzer reprocessing (Figure 1A) involves cleaning, disinfection, rinsing, and integrity testing, which require an estimated additional water consumption of approximately 5–10 L per dialyzer^15^. Lacson and Lazarus^9^ estimated 100 L for 10 units. In our unit, projecting 10 L per reprocessed dialyzer resulted in an estimated increase of 2.3% in total water use (429 L/session), indicating that the additional water demand from reprocessing was proportionally modest.

Under a single-use scenario, an estimated 163,190 additional items would have been discarded, representing a 12.6-fold increase in dialyzers and a 13-fold increase in blood tubing sets. Total waste was projected to increase by 24,319.9 kg (+78.7%), with a 312% increase in incinerated plastics. Applying an emission factor of 1.80 kg CO_2_-eq per kg of incinerated clinical waste^1^, this would result in an estimated additional emission of 37,492 kg CO_2_-eq, equivalent to the combustion of 16,100 L of gasoline^16^.

The unit generated 30.9 t of solid waste over the study period, equivalent to the weight of 31 standard passenger vehicles or 6,181,140 plastic bags of 5 g each. Mean annual waste generation was 138.6 kg/patient/year (0.89 kg/session), of which only 0.192 kg/session was non-recyclable infectious waste. Hoenich et al.^12^ reported 2.5 kg/session (390 kg/patient/year) in UK dialysis units during the 1990s under single-use practice, 2.81 times higher than observed here. In Italy, Piccoli et al.^7^ documented 1.5–8 kg of plastic waste per session across 30 evaluated sessions, also under a single-use regimen.

These data underscore the waste reduction potential of dialyzer reuse, particularly regarding incineration-related emissions. As noted by Piccoli et al.^7^, although reuse is banned in several European countries, the low quality and heterogeneity of the available evidence warrant reconsideration of this practice in the context of ecological sustainability, balancing plastic waste reduction against the use of potentially toxic chemicals in reprocessing. Current evidence supports dialyzer reuse as a viable option, particularly where resources are limited. A recent review by Thongsricome^8^ found no consistent evidence of superiority of single use over reuse for hospitalizations or mortality. According to the authors, unfavorable results associated with reuse were mainly linked to inappropriate use of peracetic acid and technical failures during reprocessing; after controlling for these variables and comorbidities, mortality differences disappeared. Similarly, Bond et al.^17^, in an analysis of more than 27,000 patients using more robust statistical methods (propensity score, instrumental variables, and time-dependent survival analysis), not only found no significant difference between the strategies but also identified a protective effect of reuse in the time-dependent analysis.

This study describes the real-world experience of a medium-sized center in northeastern Brazil, with consumption data obtained directly from the utility company, the unit’s only water source; this water undergoes treatment equivalent to that provided for human consumption. This single-center study is limited by the inability to disaggregate water consumption by specific activity (sessions, reverse osmosis, or reprocessing). Energy use, supply transport, and chemical inputs were not measured; consequently, these data constitute a partial environmental assessment rather than a full life-cycle analysis. Addressing these gaps should be a priority for future research. The single-use scenario was based on mathematical projections and literature-derived factors and is subject to uncertainty. The values should be interpreted as approximate estimates. Decisions about reprocessing must weigh patient safety, regulatory requirements, and socioeconomic context; the findings do not constitute a universal recommendation.

In this setting, dialyzer reuse was associated with substantially lower waste generation and incineration-related emissions than projected under a single-use scenario. These findings are environmental estimates; they do not support causal inferences or universal recommendations, but they do provide quantitative data to inform sustainability decisions in dialysis units operating under resource constraints.

## Data availability

The datasets generated and/or analyzed during the present study are available from the corresponding author upon reasonable request.
