# Supplementary material for: Green nephrology: water and waste — one year in a hemodialysis unit in Northeastern Brazil and reassessment of dialyzer reprocessing from an environmental perspective
Source: J Bras Nefrol. 2026 Aug 7;48(4):e20250302. doi: 10.1590/2175-8239-JBN-2025-0302en (PMC13450943; doi:10.1590/2175-8239-JBN-2025-0302en)
Supplement: Translation

# Translation: *Green nephrology*: água e resíduos – 1 ano em uma unidade de diálise no Nordeste do Brasil – repensando o reprocessamento de dialisadores à luz de estimativas ambientais

**DOI:** 10.1590/2175-8239-JBN-2025-0302pt

**Authors:** Ginivaldo Victor Ribeiro do Nascimento, Isabella Melo Soares, Elizana Carvalho Oliveira, Thiago de Sousa Coelho Porto

**Affiliations:** 1 Universidade Estadual do Piauí, Teresina, PI, Brasil.; 2 Centro Universitário UniFacid, Teresina, PI, Brasil.; 3 Centro de Terapia Renal, Teresina, PI, Brasil.

**Keywords:** Diálise Renal, Sustentabilidade Ambiental, Meio Ambiente, Reciclagem, Resíduos

## Abstract

**Introdução:**

Hemodiálise é uma das terapias de maior consumo de água e geração de resíduos, com impacto ambiental pouco documentado em países de baixa e média renda. O objetivo deste estudo foi estimar consumo de água e geração de resíduos sólidos em uma unidade de hemodiálise do Nordeste brasileiro que utiliza reuso de dialisadores, comparando-os a um cenário contrafactual estimado de uso único.

**Métodos:**

Estudo observacional retrospectivo de centro único. Foram analisados dados institucionais agregados de consumo hídrico, aquisição de insumos e massa de resíduos durante 12 meses. Um cenário contrafactual estimado de uso único foi construído por projeções matemáticas, assumindo um dialisador novo por sessão. Emissões de CO_2_ equivalente (CO_2_-eq) foram estimadas com base em fator de emissão para incineração de resíduos clínicos.

**Resultados:**

Foram realizadas 34.759 sessões de hemodiálise, com média de 223 pacientes/mês. Consumo total de água: 14,9 milhões de litros, correspondendo a 429 L/sessão (2,3% atribuídos ao reprocessamento). Geração de resíduos sólidos: 30,9 t (0,89 kg/sessão), sendo 21,6% resíduos contaminados, 51,5% recipientes de soluções/frascos, 26,8% papelão. Do total de plástico, 70,4% foram reciclados e 29,6% incinerados. No cenário contrafactual de uso único, estimou-se acréscimo de 78,7% em resíduos, incremento de 312% nos plásticos incinerados e emissão estimada adicional de 37,5 t de CO_2_-eq.

**Conclusão:**

A comparação dos dois cenários sugere que o reuso de dialisadores apresentou impacto marginal no consumo hídrico e redução na geração de resíduos e nas emissões associadas. As comparações devem ser interpretadas como estimativas ambientais, não permitindo inferência causal ou recomendação universal.

## INTRODUÇÃO

A hemodiálise é uma das terapias que mais consomem água e energia na saúde^1,2^. O impacto ambiental desse tratamento ganhou destaque apenas nos últimos anos, acompanhando o crescimento global da doença renal crônica. Estima-se que, em 2025, o número de pacientes em diálise alcance 5-6 milhões, com prevalência de 824 pmp^3,4^.

Em muitas regiões, a escassez de água já é uma realidade, e a gestão dos recursos hídricos em hemodiálise é negligente, sobretudo pela osmose reversa, que descarta até dois terços do insumo^3,5^. Outros fatores relacionados à diálise e às prescrições de diálise também influenciam o consumo de água^3,5^.

A geração de resíduos é outro desafio. Menos de um terço dos não infectantes é reciclado, enquanto os infectantes, destinados à incineração, contribuem para a emissão de CO_2_ e para o agravamento das mudanças climáticas^3,6,7^. Nesse contexto, o reprocessamento de filtros dialisadores surge como alternativa potencial, reduzindo a poluição e a geração de resíduos, sem que haja evidências consistentes de inferioridade clínica^7,8^. Ainda assim, a maioria das publicações disponíveis apresenta apenas análises parciais, sem detalhar de forma sistemática a composição dos resíduos gerados e seu destino final. O conhecimento aprofundado sobre recursos, insumos e resíduos é fundamental para embasar estratégias de mitigação e promover uma destinação sustentável.

O objetivo deste estudo foi avaliar o consumo de água e a geração de resíduos ao longo de 12 meses em uma clínica de hemodiálise no Nordeste do Brasil, comparando-os com um cenário estimado de uso único de filtros dialisadores.

## MÉTODOS

Estudo observacional retrospectivo, realizado em centro único: unidade de hemodiálise privada conveniada ao Sistema Único de Saúde (SUS), com 98,3% dos pacientes com financiamento público, localizada em Teresina, Piauí. A unidade opera em três turnos diários de diálise, exceto aos domingos, utilizando reuso de dialisadores e linhas de sangue, realizado por sistema automatizado. Foram analisados os dados de 1° de agosto de 2022 a 30 de julho de 2023.

O consumo de água foi obtido junto à companhia de abastecimento, única fornecedora da unidade. O volume anual incluiu sessões de hemodiálise, sistema de osmose reversa, limpeza e desinfecção dos equipamentos, reprocessamento e demais atividades operacionais, não sendo possível discriminar o consumo por atividade específica. Para estimar o impacto hídrico do reprocessamento, considerou-se um consumo adicional aproximado de 10 L de água por filtro reutilizado, conforme descrito na literatura, reconhecendo-se a possível variação operacional^9^.

As informações sobre insumos foram obtidas a partir dos registros da farmácia/almoxarifado. As embalagens foram pesadas e classificadas por tipo, incluindo filtros dialisadores, embalagens plásticas e recipientes plásticos rígidos utilizados para armazenamento de soluções concentradas. Os cálculos referentes ao cenário observado basearam-se no número total de filtros dialisadores efetivamente consumidos no período analisado, conforme registros institucionais, não tendo sido adotado um número médio fixo de reutilizações como parâmetro de modelagem. Embora o protocolo institucional permita até 20 reutilizações por unidade, essa variável não foi empregada diretamente nas estimativas ambientais do presente estudo. Os dados relativos aos resíduos não recicláveis foram fornecidos pela empresa de gerenciamento contratada.

Além do cenário observado, foi construído um cenário contrafactual estimado de uso único, assumindo a utilização de um filtro dialisador e um conjunto de linhas de sangue novos por sessão, mantendo-se o mesmo número total de sessões realizadas no período. Assim, o número total projetado de filtros e linhas no cenário de uso único foi considerado equivalente ao número total de sessões, sendo o quantitativo adicional estimado pela diferença entre o total de sessões e o número efetivamente consumido no cenário observado com reuso. As estimativas foram obtidas por projeções matemáticas e não correspondem a medições empíricas diretas.

As emissões de dióxido de carbono equivalente (CO_2_-eq) foram estimadas multiplicando-se a massa de resíduos incinerados por um fator de emissão de 1,80 kg CO_2_-eq/kg de resíduo^1^.

O número de pacientes foi registrado mensalmente para cálculo da média anual. O consumo médio de água e de resíduos por paciente/ano foi calculado com base no total de sessões. Os dados contínuos foram expressos em totais e médias, relativos por paciente ou sessão, e os categóricos, em números absolutos e percentuais.

Por tratar-se de estudo baseado exclusivamente em dados institucionais agregados, sem identificação individual ou acesso a informações nominativas, o projeto foi considerado dispensado de submissão ao Comitê de Ética em Pesquisa, nos termos da Resolução CNS nº 510/2016. Não houve necessidade de obtenção do Termo de Consentimento Livre e Esclarecido (TCLE), por não envolver dados individualizados.

## RESULTADOS

Foram realizadas 34.759 sessões de hemodiálise ao longo de 12 meses, correspondentes ao tratamento de uma média de 223 pacientes/mês. O consumo total de água foi de 14.920.000 L, e o consumo médio correspondeu a 66.906 L/paciente/ano e 429 L/sessão (Tabela 1).

**Tabela 1:** Consumo de água, geração de resíduos e emissões estimadas de CO_2_ em clínica de diálise no nordeste do brasil: comparação entre uma clínica com reuso de dialisadores e um cenário estimado com uso único de dialisadores (12 meses)

Métrica	Reuso (observado)	Uso único* (estimado)	Diferença (uso único vs. reuso)
Total de água (12 meses, L)	14.920.000	14.572.410	−2,3%
Água média por sessão (L/sessão)	429	419	−2,3%
Total de resíduos (kg/ano)	30.905,7	55.225,1	+78,7%
Total reciclado (%) kg/ano	24.223,2 (78,4)	27.714,2 (50,2)	+14,4%^δ^
Plásticos reciclados (kg/ano)	15.920,2	15.920,2	0%
Papelão reciclado (kg/ano)	8.304	11.794	+42%
Total incinerado (%) kg/ano	6.682,5 (21,6)	27.510,9 (49,8)	+312%
Unidades plásticas incineradas (no de itens)	99.092	163.190	+64,7%
Emissões de CO_2_ * (kg de CO_2_-eq/ano)	12.028,5	49.535,8	+312%

Nota – *Valores estimados

^δ^ = diferença na massa

A geração de resíduos sólidos alcançou 30.905,7 kg. Desse total, 21,6% eram resíduos contaminados destinados à incineração, incluindo plásticos como filtros dialisadores, linhas de sangue e agulhas; 51,5% foram compostos por recipientes plásticos para armazenamento de soluções concentradas e frascos de soro; e 26,8% por caixas de papelão. A média anual por paciente foi de 138,6 kg (0,89 kg/sessão).

Quanto ao número de unidades plásticas, 57,9% foram encaminhadas à reciclagem (136.724 unidades de frascos de soro e recipientes plásticos de soluções) e 42,1% à incineração, correspondendo a 57.258 agulhas, 2.668 linhas de sangue, 2.752 filtros e 36.414 equipos. Em massa, 70,4% dos resíduos plásticos foram reciclados (15.920,2 kg), e 29,6%, incinerados. O papelão reciclado somou 8.304 kg, reaproveitado na indústria de detergentes automotivos, enquanto as embalagens de frascos de soro foram enviadas para trituração e reutilizadas como matéria-prima para novas embalagens plásticas (Tabela 1; Figura 1B–D).

**Figura 1.** (A) Unidade de reprocessamento de dialisadores. (B) Trituramento de resíduos plásticos usados na clínica de hemodiálise. (C e D) Transformação de resíduos plásticos em matéria-prima para a criação de novos produtos plásticos.

No período, a unidade gerou 30,9 t de resíduos, dos quais apenas 0,192 kg/sessão correspondiam a resíduos infectantes não recicláveis.

## DISCUSSÃO

Segundo o Censo Brasileiro de Diálise de 2024, o Brasil tem 172.585 pacientes em hemodiálise, sendo 3.412 no Piauí, um dos estados de menor desenvolvimento socioeconômico, distribuídos em 15 centros^10,11^. Nessa realidade, marcada por restrições orçamentárias, são pouco frequentes práticas de reciclagem de água ou de uso único de filtros dialisadores, predominando o reprocessamento automatizado. Assim, os achados deste estudo refletem não apenas uma prática local, mas também a realidade de uma parcela expressiva dos serviços de diálise do país.

A crescente atenção internacional às mudanças climáticas e à sustentabilidade dos serviços de saúde enfatiza o consumo de água e a geração de resíduos como pontos críticos para a hemodiálise^1,2,3,5,6,7,12,13^. Neste estudo, verificou-se consumo anual de 14,9 milhões de L de água em uma única unidade, equivalente ao volume necessário para encher seis piscinas olímpicas ou ao abastecimento anual de uma cidade de quase 1.000 habitantes (Figura 2). O consumo médio observado (429 L/sessão) foi superior ao relatado por Sahay et al.^14^ (250 L) e semelhante a estimativas amplamente utilizadas na literatura (500 L), as quais derivam de cálculos teóricos, considerando fluxo de dialisato de 500 mL/min, por quatro horas de sessão, acrescido da perda relacionada à osmose reversa^3,6,14^. No presente estudo, o valor anual de 66.906 L/paciente foi inferior à projeção teórica de 80.000 L^3,8,14^.

**Figura 2.** Água, resíduos e emissões de CO_2_ – 1 ano em uma clínica de hemodiálise no Nordeste do Brasil.

O reprocessamento de dialisadores (Figura 1A) envolve etapas de limpeza, desinfecção, enxágue e testes de integridade, que implicam consumo adicional de água estimado em cerca de 5–10 L adicionais por filtro^15^. Lacson e Lazarus^9^ estimaram 100 L para 10 unidades. Dessa forma, na clínica estudada, projetou-se acréscimo de 10 L por unidade reprocessada, correspondendo a um aumento estimado de 2,3% no volume total (429 L/sessão), o que sugere impacto hídrico proporcionalmente pequeno em relação à redução de resíduos observada.

Por outro lado, caso a unidade de diálise estudada realizasse uso único de filtros dialisadores e linhas de sangue, estima-se descarte de 163.190 insumos adicionais, aumento de 12,6 vezes no número de filtros dialisadores e de 13 vezes no número de linhas de sangue. Isso resultaria em acréscimo de 24.319,9 kg de resíduos (+78,7%), com incremento de 312% no volume de plásticos incinerados. Adotando-se o fator de emissão de 1,80 kg de CO_2_-eq por kg de resíduo clínico incinerado^1^, haveria emissão estimada adicional de 37.492 kg de CO_2_-eq, equivalente à combustão de 16,1 mil L de gasolina^16^.

No período avaliado, a unidade gerou 30,9 t de resíduos sólidos, equivalente ao peso de 31 automóveis populares ou a 6.181.140 sacolas plásticas de 5 g. A média por paciente foi de 138,6 kg/ano (0,89 kg/sessão). Apenas 0,192 kg/sessão correspondiam a resíduos infectantes não recicláveis. Hoenich et al.^12^ relataram, no Reino Unido, na década de 1990, período em que já se adotava uso único de filtros dialisadores naquela nação, geração de 2,5 kg de resíduos/sessão, dos quais 38% eram plásticos, totalizando 390 kg/paciente/ano, 2,81 vezes acima do observado neste estudo. Na Itália, Piccoli et al.^7^ documentaram 1,5 a 8 kg de resíduos plásticos por sessão ao longo de 30 sessões avaliadas, também em regime de uso único.

Esses achados reforçam a necessidade de considerar o reuso sob o ponto de vista do impacto ambiental, especialmente em termos de redução de resíduos e de emissões associadas à incineração. Conforme Piccoli et al.^7^, embora o reuso seja banido em vários países europeus, a baixa qualidade e a heterogeneidade das evidências disponíveis tornam oportuno repensar essa prática à luz da sustentabilidade ecológica, equilibrando a redução de resíduos plásticos com o uso de produtos químicos potencialmente tóxicos no reprocessamento. A literatura disponível até o momento indica que o reuso de dialisadores permanece uma opção válida, sobretudo em locais com recursos limitados. Uma revisão recente de Thongsricome^8^ não encontrou evidências consistentes de superioridade do uso único sobre o reuso em desfechos como hospitalizações e mortalidade^8^. Segundo os autores dessa publicação, resultados desfavoráveis ao reuso foram associados principalmente ao uso inadequado de ácido peroxiacético e a falhas técnicas no reprocessamento; após controle para essas variáveis e para comorbidades, as diferenças de mortalidade desapareceram^8^. De forma semelhante, Bond et al.^17^, em análise de mais de 27.000 pacientes com métodos estatísticos mais robustos (escore de propensão, variáveis instrumentais e análise de sobrevida dependente do tempo), não apenas não encontraram diferença significativa entre as estratégias, como identificaram efeito protetor do reuso na análise dependente do tempo.

O presente estudo retrata um cenário de vida real de um centro de médio porte no Nordeste do Brasil, com dados de consumo obtidos diretamente da concessionária, sendo esta a única fonte utilizada pela unidade, cuja água passa por tratamento equivalente à água dispensada para consumo humano. Limitações incluem análise restrita a um único centro; o consumo hídrico foi obtido como volume total institucional, não sendo possível discriminar o consumo específico das sessões, da osmose reversa ou do reprocessamento. Além disso, não foram mensurados consumo energético, transporte de insumos ou uso de produtos químicos. Assim, esta análise representa uma avaliação parcial do impacto ambiental, não configurando uma análise de ciclo de vida completa. Esses componentes constituem agenda prioritária para estudos futuros. O cenário de uso único baseia-se em projeções matemáticas e fatores da literatura, estando sujeito a incertezas. Os valores devem ser interpretados como estimativas aproximadas. Decisões sobre reuso devem considerar simultaneamente segurança do paciente, regulação sanitária e contexto socioeconômico, não sendo possível extrapolar os resultados como recomendação universal.

Em conclusão, o reuso de dialisadores esteve associado à menor geração de resíduos no cenário observado. As projeções contrafactuais sugerem que o uso único poderia aumentar a massa de resíduos e as emissões associadas. Esses achados devem ser interpretados como estimativas ambientais, não permitindo inferências causais definitivas ou recomendações universais.

## Disponibilidade de dados

Os conjuntos de dados gerados e/ou analisados durante o presente estudo estão disponíveis mediante solicitação razoável ao autor correspondente.
